# PEGylated pUR4/FUD peptide inhibitor of fibronectin fibrillogenesis decreases fibrosis in murine Unilateral Ureteral Obstruction model of kidney disease

**DOI:** 10.1371/journal.pone.0205360

**Published:** 2018-10-24

**Authors:** Bianca R. Tomasini-Johansson, Pawel W. Zbyszynski, Inger Toraason, Donna M. Peters, Glen S. Kwon

**Affiliations:** 1 School of Pharmacy, University of Wisconsin, Madison, Wisconsin, United States of America; 2 Pathology and Laboratory Medicine, School of Medicine and Public Health, University of Wisconsin, Madison, Wisconsin, United States of America; Center for Molecular Biotechnology, ITALY

## Abstract

Fibronectin is a blood and extracellular matrix glycoprotein that plays important roles in wound healing and fibrosis since it controls the deposition of collagen and other extracellular matrix molecules and is a substrate for infiltrating lymphocytes. Using a high-affinity fibronectin-binding peptide (FUD/pUR4) that inhibits fibronectin deposition into extracellular matrix (ECM), we tested the ability of a PEGylated FUD/pUR4 (PEG-FUD) to inhibit fibrosis in the Unilateral Ureteral Obstruction (UUO) kidney disease model. Fibronectin fibrillogenesis assays, using human fibroblasts and human proximal tubular epithelial cultures, showed that PEG-FUD can inhibit fibronectin fibrillogenesis in vitro with an IC50 similar to unconjugated FUD, in the order of 20–35 nM. In contrast, a mutated FUD (mFUD) conjugated to PEG that lacked activity did not inhibit fibronectin assembly, even at 20 μM. The in vivo activity of PEG-FUD was tested in the murine UUO model by daily subcutaneous injection of 12.5 mg/kg for 7 days until harvest at day 10. Control treatments included saline, PEG, unconjugated FUD, and PEG-mFUD. Immunoblotting studies showed that fibronectin was enriched in the extracellular matrix fractions of extracted UUO kidneys, compared to contralateral untreated kidneys. In vivo, PEG-FUD significantly decreased fibronectin by ~70% in UUO kidneys as determined by both IHC and immunoblotting, respectively. In contrast, neither PEG-mFUD, PEG, nor saline had any significant effect. PEG-FUD also decreased collagens I and III and CD45-expressing cells (leukocytes) by ~60% and ~50%, as ascertained by picrosirius red staining and IHC, respectively. Immunoblotting studies also showed that the fibronectin remaining after PEG-FUD treatment was intact. Utilizing a custom-made polyclonal antibody generated against pUR4/FUD, intact PEG-FUD was detected by immunoblotting in both the ECM and lysate fractions of UUO kidneys. No adverse reaction or event was noted with any treatment. In summary, these studies suggest that PEG-FUD reached the kidneys without degradation, and decreased fibronectin incorporation into interstitial tissue. Decreased fibronectin was accompanied by a decrease in collagen and leukocyte infiltration. We propose that PEG-FUD, a specific inhibitor of fibronectin assembly, may be a candidate therapeutic for the treatment of fibrosis in kidney diseases.

## Introduction

Approximately 30 million people in the U.S. suffer from some level of chronic kidney disease. The therapeutic interventions, such as medications that decrease blood pressure, are not entirely effective as approximately 660,000 patients proceed to develop End Stage Renal Disease (ESRD). Dialysis and transplantation are treatment options for ESRD but only about 17% of those in need receive a transplant and the 10-year mortality rate for dialysis patients is ~65%. Following transplantation, there is a 5 and 10 year graft failure rate of approximately 30% and 50%, respectively [1, 2]. Both kidney disease and eventual graft failure are associated with fibrosis, which is characterized by excess connective tissue deposition and, in some circumstances, inflammation [3–6]. Fibronectin is a blood and extracellular matrix (ECM) glycoprotein that controls the deposition of a number of other ECM proteins, including collagens and latent TGF-β binding protein [7–10], and the attachment of inflammatory lymphocytes [11]. Fibronectin also activates lysyl oxidase, increasing collagen cross-linking and thereby increasing matrix stiffness [12]. Elevated levels of fibronectin in fibrotic kidneys have been demonstrated in various models of kidney disease [13–15], but the effects of specific inhibition of fibronectin deposition have not been reported.

We previously developed a polypeptide inhibitor termed pUR4/FUD that specifically binds to N-terminal region of fibronectin and blocks its incorporation into the ECM, in turn reducing collagen deposition [8, 16]. pUR4/FUD was reported to successfully reduce fibrosis in murine models of coronary artery disease, experimentally induced liver fibrosis [17, 18], and more recently, heart failure [19]. In order to avoid the difficulties inherent in administering peptides in vivo, including rapid clearance in the kidney, as well as to take advantage of possible protection against peptide degradation and longer circulation time [20, 21], we PEGylated pUR4/FUD specifically at the N-terminus with polyethylene glycol (PEG) of 20,000 g/mol and characterized its binding to fibronectin, determining that PEGylation did not affect affinity of pUR4/FUD towards fibronectin [22]. In this work, we investigated whether PEG-pUR4/FUD reaches the kidneys and inhibits fibronectin deposition and fibrosis in the unilateral ureteral obstruction (UUO) model of kidney disease. Hereafter, pUR4/FUD will be referred to as FUD.

## Materials and methods

### Preparation of PEGylated peptides

FUD and a mutated version of FUD (mFUD, does not inhibit fibronectin fibrillogenesis) were generated and characterized as previously reported [23, 24]. Both PEG-FUD and the control PEG-mFUD were generated and characterized as described [22]. The concentration of FUD and PEGylated FUD was obtained from absorbance measurements at 280 nm, using ε = 0.496, as described previously [23]. The concentration of PEG-mFUD was ascertained using A280 values and ε = 0.744. Both PEG-FUD and PEG-mFUD were dosed in peptide mg equivalents. PEG (20,000 kDa) was administered on a weight/volume basis. Purification of plasma fibronectin and its conjugation to Alexa 488 was performed as previously described [25].

### Microtiter fibronectin matrix assembly assays

#### Exogenous plasma fibronectin matrix assembly assay

Binding and incorporation of Alexa488-plasma fibronectin (A488-FN) to fibroblast monolayers (human foreskin fibroblasts, AH1F cells [26] was carried out as described [25]. Briefly, fibroblasts (60000 per well) were allowed to adhere and spread for 2 hours in microtiter plates in 2% fetal calf serum (FCS)-containing DMEM. Peptides or modulators to be tested were added followed by 28 nM A488-FN. Cell monolayers with or without peptides and A488FN were incubated overnight at 37^°^C with 5% CO_2_. Cell monolayers in microwells were washed with PBS or HBSS containing Ca^2+^, Mg^2+^. Fluorescence in 96-well plates was read using a microplate reader using bottom-readout capabilities at 485 nm excitation and 525 nm emission with 20 nm bandwidths. To estimate cell viability per well after the washes and measurement of fluorescence, an equal volume of luminescence reagent (Cell Titer Glo) was added followed by quantification of luminescence. Fluorescence of wells to which A488FN had not been added, i.e. intrinsic fluorescence of the cell monolayer was subtracted from fluorescence values of wells containing ligand. Data is presented respectively for fibronectin fibrillogenesis or cell viability as percent of fluorescence or luminescence values for wells with no peptides. Microplate microscopy was used to ascertain that fluorescence measurements correspond to fibrillary fibronectin, as previously demonstrated [25].

#### Endogenous (cellular) fibronectin assembly assay

This immunochemical assay measures the ECM incorporation of EDA-containing fibronectin synthesized by human fibroblasts and human proximal tubular epithelial cells [27]. Human proximal epithelial cells, HK-2 cells, were obtained from ATCC (CRL-2190). The EDA+ fibronectin is a splice variant synthesized and secreted by a variety of cells mostly during embryonic development and during wound healing, ECM remodeling, or fibrotic conditions in the adult [28–30]. Fibroblasts (AH1F) were cultured in DMEM (Gibco) containing 10% fetal calf serum, and human proximal tubular epithelial cells (HK-2) were cultured in keratinocyte serum free medium (KSFM, containing pituitary extract and rEGF, Invitrogen/Thermo Fisher). Both media also contained 2.5 mM glutamine, 100 IU/ml penicillin, and 100 μg/ml streptomycin. Confluent cells were trypsinized and resuspended in DMEM containing 2% FCS in DMEM for AH1F cells or KSFM for HK-2 cells, at densities that allow delivery of 60,000 AH1F or 20,000 HK-2 cells in 90 μl per well in black-walled, flat transparent bottom 96-well plates. Cells were incubated for 2 h at 37^°^C with 5% CO_2_ and checked microscopically for spreading and confluence. Peptides were then added in 10 μl volumes at 10-fold the desired final concentration. Cell monolayers in microplates were incubated overnight in a humidified 37^°^C, 5% CO_2_ chamber. After approximately 22 h, 10 μl of Alamar Blue was added to the cell monolayers and incubated at 37^°^C for 2 h to assess cell viability. Fluorescence of Alamar Blue was read at 555 nm excitation and 585 nm emission. Microplates were washed twice with HBSS containing Ca^2+^, Mg^2+^ and 50 μl/ well of 4% paraformaldehyde (PFA) in PBS was then added for 10 min at room temperature. Following two PBS washes, 100 μl 2% BSA/PBS was added for 1 h at room temperature to block nonspecific binding. The BSA solution was removed and Alexa488-labeled anti cellular FN monoclonal antibody (A488-EDA, eBioscience/Thermo Fisher cat. No. 53-9869-82) or a control antibody, Alexa 488-labeled IgG1, diluted in 2% BSA/PBS to 2.5 μg/ml was added and incubated for 1 h at room temperature. Similar to the measurement of exogenous A488-FN fluorescence, A488-EDA fluorescence was read using microplate reader equipped with bottom-readout capabilities at 485 nm excitation and 525 nm emission. Fluorescence of wells to which A488-IgG was added, i.e. background green fluorescence from non-specific IgG binding and from the cell monolayer was subtracted from fluorescence values of wells incubated with A488-EDA. Data is presented for fibronectin fibrillogenesis or cell viability as percent of fluorescence values (A488 fluorescence or Alamar Blue, respectively) for wells with no peptides.

#### Unilateral ureteral obstruction model

Male C57BL/6 mice were purchased from Envigo (Madison, WI) and housed in the Wisconsin Institutes for Medical Research animal facilities at the University of Wisconsin-Madison with ad-libitum access to food and water. Animals were maintained in humidity and temperature-controlled rooms under 12 h light/dark cycles. All work was conducted under protocol M5421, reviewed and approved by the University of Wisconsin-Madison Institutional Animal Care and Use Committee. All efforts were made to minimize suffering. The Unilateral Ureteral Obstruction (UUO) model is a rodent surgical model representing a human equivalent of acute kidney injury [14, 31–33]. Obstruction results in marked dilatation of the ureter together with reduced renal blood flow and glomerular filtration. Renal histology demonstrates tubular atrophy and increasingly severe interstitial renal inflammation and fibrosis. UUO or sham surgeries were performed as previously described [33] on 10-week male C57BL/6 mice weighing approximately 20–23 g following acclimation to the vivarium facilities. Briefly, under 2% isoflurane anesthesia, the left kidney and ureter were exposed through a midline or a flank incision. The ureter was ligated using black braided 7-O silk suture material. The ligated ureter and kidney were returned to the abdominal cavity and the incision was closed in two layers with interrupted sutures and Vet Bond tissue adhesive or staples. The right or contralateral kidney was used as a control. Sham operated animals were treated as the UUO animals except their ureter was not ligated. Animals were given ketoprofen (5 mg/kg) prior to returning them to their cages where they were kept on standard water and chow until sacrifice at the designated times. Peptides, PEGylated peptides or PEG were diluted in physiological saline, prepared for in vivo administration as described previously [17], and administered subcutaneously at 0.3 mg per day (~12.5 mg/kg). PEGylated peptide equivalent amounts were based on peptide concentration. Treatment injections were started three days following surgery and continued daily until 24 h before harvest at day 10. At harvest, under isoflurane, blood was collected from the aorta and both UUO and contralateral kidneys removed. Each kidney was sectioned and one half placed in 10% formalin for 24 h, stored in 70% ethanol until processing for embedding in paraffin, sectioning and histological staining. The other half of each kidney was trimmed to remove the medulla and frozen in liquid nitrogen until extraction for Western blotting.

Sham surgeries in which the left ureter was handled but not tied resulted in levels of fibronectin or collagen comparable to contralateral kidneys. Thus, the right, contralateral, kidney of each mouse was utilized as a control to the left UUO-treated kidney. The data from two cohorts of UUO-treated mice were combined for this study composed of the following groups (n): saline (n = 5), PEG (n = 4), FUD (n = 5), PEG-FUD (n = 8) and PEG-mFUD (n = 3).

#### Histology and immunostaining

Paraffin-embedded tissues were sectioned at 4 μm and stained with H&E and Picrosirius Red, and IHC for fibronectin and CD45 for leukocytes. Picrosirius red staining is specific for collagens I and III and is considered to be superior to trichrome staining for quantitation of collagens [14, 34, 35]. Staining for fibronectin was carried out using a rabbit polyclonal antibody to mouse fibronectin (RamFN) which was previously described [36]. RamFN diluted at 1:5000 required prior proteinase K antigen retrieval and was developed with ImmPRESS Rabbit Ig HRP. Staining for leukocytes was carried out using the pan leukocyte antibody rat anti-mouse CD45 (Leukocyte Common antigen, Ly-5, BD Pharmingen, clone 30-F11) at 1:100 after citrate pH 6.0 antigen retrieval and developed with ImmPRESS rat Ig HRP. RamFN and CD45 staining was counter stained with hematoxylin to demarcate nuclei.

Stained tissue sections were imaged using the 20 X objective on an upright brightfield Nikon Eclipse 600 microscope. This microscope was also equipped with polarizer capabilities utilized to assess the birefringence of the picrosirius red-stained collagen fibers. Quantitation of birefringence or DAB product following development of HRP stains was carried out using Image J (Sun Microsystems.Ink) from 6 images from each mouse section obtained at random within the central cortical area and excluding large blood vessels and the kidney capsule.

### Tissue extraction and immunoblotting

#### ECM and lysate/membrane fractions from kidney extracts

Kidney tissues were fractionated as described [37]. Tissues were homogenized in RIPA buffer containing 1% deoxycholate (DOC) at 0.1g tissue per ml buffer, spun at 4^°^C and the resulting pellets were resuspended in buffer containing 4M urea, 4% SDS and 1 mM DTT. The DOC-soluble supernatant constitutes the cytosolic/membrane fraction (lysates). Resuspended pellets were vortexed and heated to 95^°^C for 5 min. This latter pool constitutes the tissue fraction containing mostly ECM proteins [18, 38, 39] with some nuclear components. The protein concentration in the ECM fraction was obtained using the DC Protein Assay kit (Bio-Rad) and albumin standards diluted in the corresponding buffer. ECM fractions (pellets) or 1% DOC-soluble fractions (lysates) were run on 4–15% or 4–20% gradient gels SDS-PAGE (Bio-Rad) at 10 μg/well, transferred to nitrocellulose and incubated with antibodies to fibronectin (RamFN) or to FUD followed by HRP-conjugated anti-rabbit (LifeTech/Thermo, Waltham, MA, USA). Relative quantitation of specific blotted protein was performed by assessing band intensities using Image J and normalizing to bands obtained with rabbit anti-Histone 3 rabbit IgG (CellSignalTechnology, Danves,MA, USA) or goat anti-GAPDH-HRP conjugated IgG (Genscript, Piscataway, NJ, USA), which were used as loading controls for ECM or lysate fractions, respectively. Alternatively, for comparison of protein levels in UUO vs contralateral tissues, we utilized Ponceau Red staining of blotted proteins prior to blocking. Images of all proteins per lane were captured, intensities measured by Image J, and utilized as loading control for normalization. SuperSignal West Femto for Maximum Sensitivitiy (ThermoFisher) was used as substrate for HRP-conjugated secondary antibodies used in Western blotting.

#### Antibody to FUD

Rabbit polyclonal antibody to FUD was custom-generated by Biomatik (http://www.biomatik.com). The antigen used for rabbit immunization consisted of synthetic preparation of the C-terminal half of FUD/pUR4 coupled to KLH via an additional cysteine residue at the N-terminus: C-DKKLPNETGFSGNMVETEDTKA. A portion of the resulting antiserum against FUD was purified by affinity chromatography on the synthetic peptide antigen coupled to an affinity matrix by the manufacturer. The titer of the affinity purified IgG was 1:128000 by direct ELISA (Biomatik). We have assessed this affinity purified antibody by Western blotting and use it at 0.7 μg/ml to assess reactivity with purified PEG-FUD and to ascertain its presence in kidney tissue extracts.

### Statistics

Graphpad Prism software was used to determine significant differences among treatment groups analyzing with Student t-test (unpaired, parametric, 2-tailed, without correction.). Probability (p) ≤ 0.05 was considered significant.

## Results

### Comparison of PEG-FUD with FUD as inhibitors of fibronectin assembly in vitro

**Fig 1:**
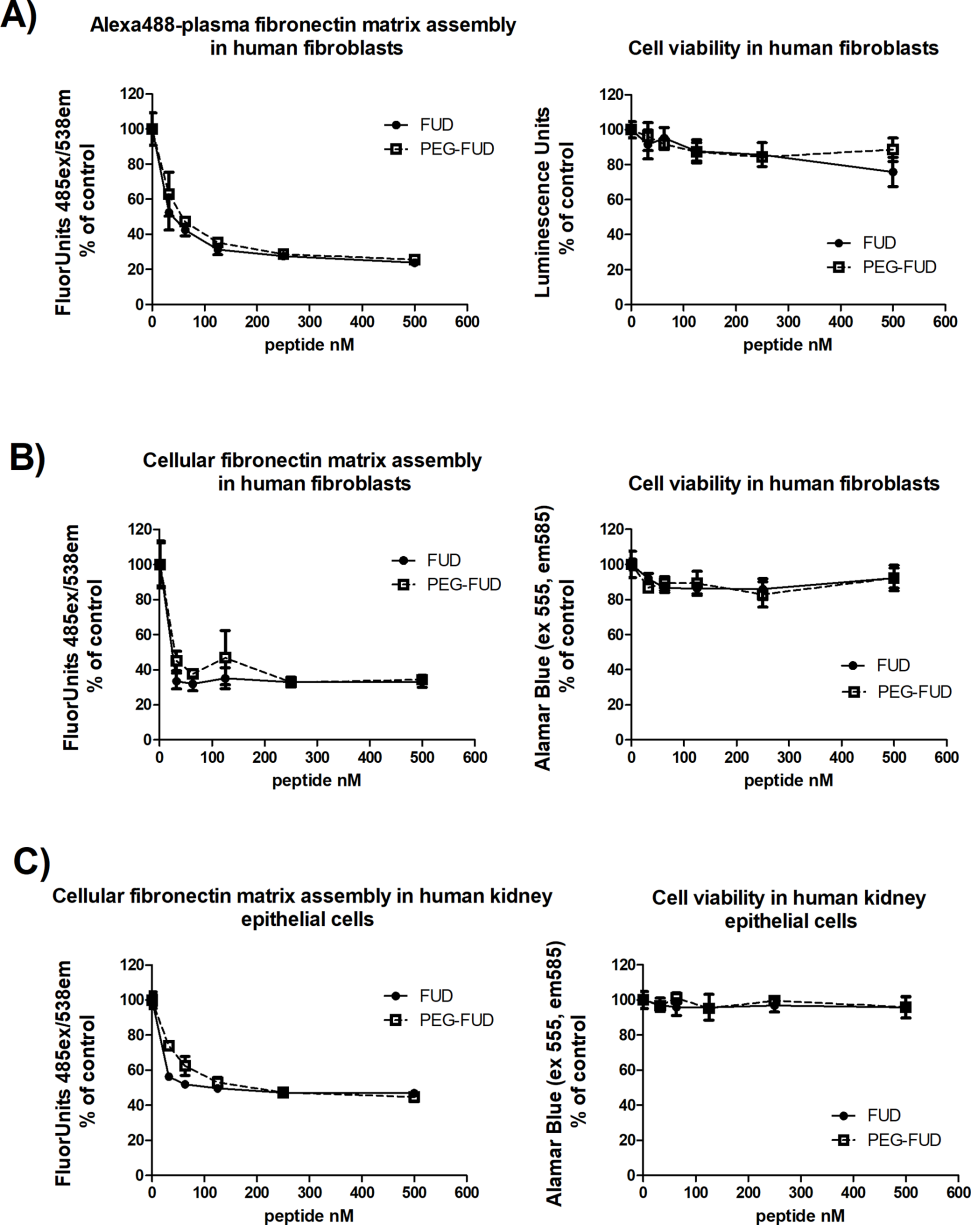
PEGylated FUD retains the ability of FUD to inhibit plasma and cellular fibronectin fibrillogenesis by human fibroblasts and proximal tubular epithelial cells in vitro. Microtiter assays measuring fluorescence associated with incorporation of fibronectin into ECM (left panels) and corresponding cell viability (right panels) following doses of FUD and PEG-FUD up to 500 nM. A) Human fibroblast incorporation of A488-plasma fibronectin; B) human fibroblast and C) human proximal tubular epithelial cell incorporation of cellular fibronectin recognized with A488-anti-EDA mAb. Data were collected in triplicate per dose and is depicted as mean +/- SD.

To determine whether PEG-FUD retained its ability to inhibit fibronectin fibrillogenesis, we compared PEG-FUD and FUD in cell-based fibronectin matrix assembly assays [25, 27]. We compared the ability of PEG-FUD to inhibit the incorporation of exogenous fibronectin derived from plasma and also of fibronectin endogenously produced by the cultured cells, since fibrosis is likely to contain fibronectin from both sources [40, 41]. Shown in Fig 1A is the level of fibrils formed from exogenously added A488-FN fibrils in the presence of increasing amounts FUD or PEG-FUD. Panels in Fig 1B and 1C show endogenous EDA+-fibronectin fibrillogenesis in human fibroblast and human proximal tubular epithelial cells, respectively. In all fluorescence panels, it is evident that PEG-FUD is as efficient as FUD at inhibiting fibronectin assembly dose-dependently with an IC50~30 nM. As previously reported [8, 16, 23, 42], cell viability is negligibly affected by FUD, and PEG-FUD behaves almost identically. There was approximately 20% decrease in fibroblast viability at the highest concentration of FUD tested (500 nM) with PEG-FUD retaining fibroblast viability almost at 100%. Both FUD and PEG-FUD were more potent inhibitors of assembly of plasma and cellular fibronectin by fibroblasts (70% inhibition) than by proximal tubular epithelial cells (50% inhibition). Human proximal tubular epithelial cell viability was retained at ~100% with treatment by both FUD and PEG-FUD. Thus, PEGylation of FUD resulted in a conjugate with fibronectin fibrillogenesis inhibitory capacity similar to that of unconjugated FUD, as demonstrated for both human fibroblasts and human proximal tubular epithelial cells.

### Testing PEG-mFUD as control peptide conjugate at high concentrations

**Fig 2:**
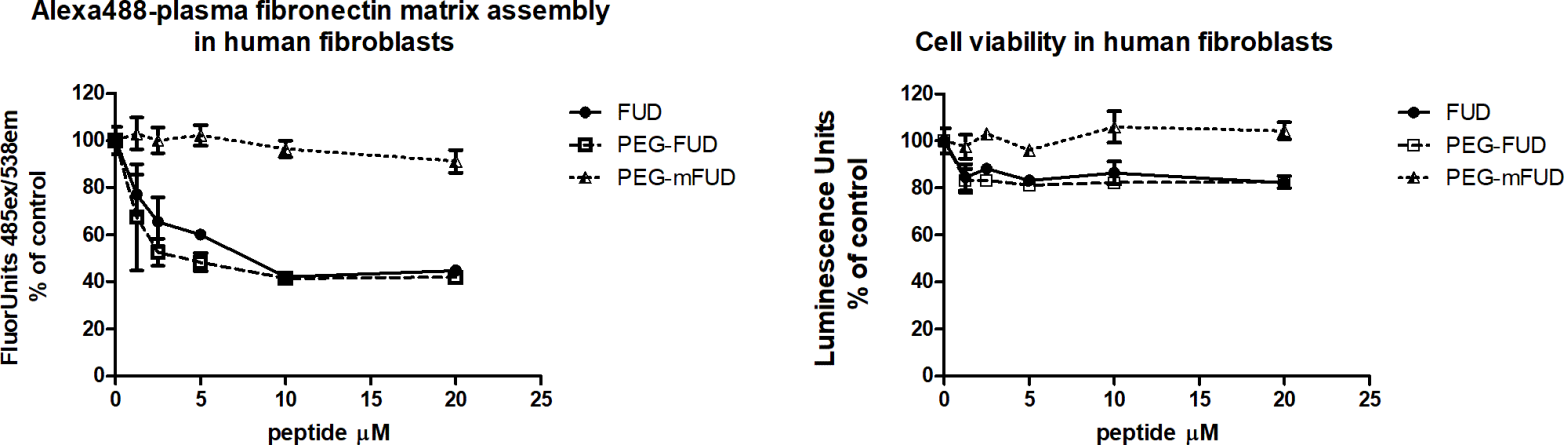
Control peptide, PEG-mFUD, does not inhibit plasma fibronectin fibrillogenesis by human fibroblasts in vitro. Microtiter assay measuring fluorescence associated with A488-plasma fibronectin into ECM (left panel) and corresponding cell viability (right panel) following doses of FUD, PEG-FUD and PEG-mFUD up to 20 μM, approximating the dose used in vivo. Data was collected in triplicate per dose and is depicted as mean +/- SD.

Having established the suitability of PEG-FUD as inhibitor of FN fibrillogenesis, we proceeded with testing a mutated form of FUD, mFUD, as a control peptide [24] in our system. mFUD was PEGylated as described above and the conjugate tested in the exogenous A488-FN binding assay as described for Fig 1A. Shown in Fig 2, concentrations of PEG-mFUD up to 20 μM did not affect fibronectin fibrillogenesis, whereas PEG-FUD and FUD behaved as expected, inhibiting fibrillogenesis by 60%, with similar IC50s in the 25–30 nM range. The right panel in Fig 2 shows cell viability remained stable at 80% at doses of PEG-FUD and FUD ranging from 1 to 20 μM, while even at the highest concentration used, PEG-mFUD had no effect on cell viability. A maximum concentration of 20 μM was tested because it approximates the concentration used in vivo, both in this study and in the liver fibrosis study [18].

### Assessment of PEG-FUD in kidney tissue extracts

**Fig 3:**
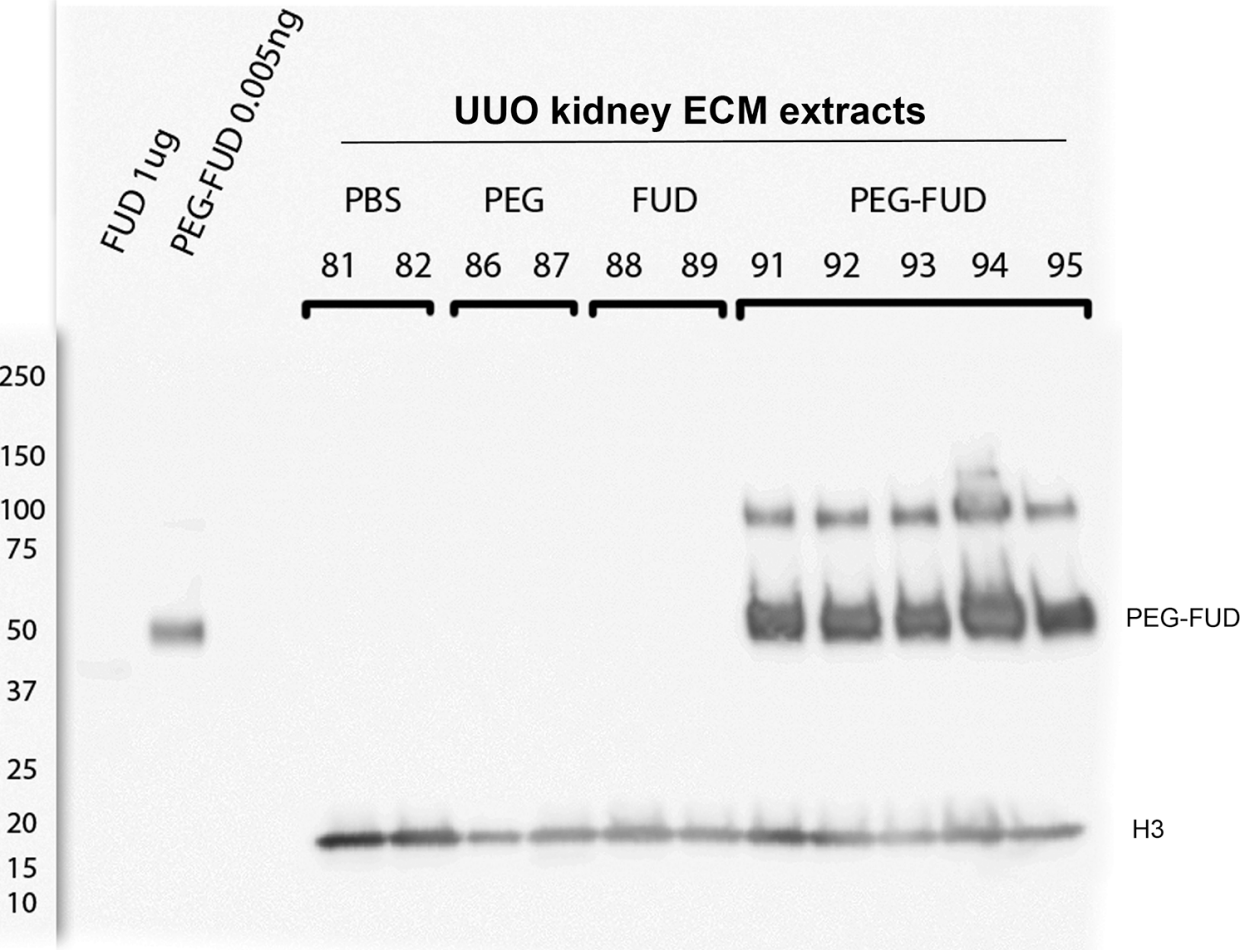
Subcutaneously administered PEG-FUD is found intact in UUO kidneys, recognized by anti-FUD polyclonal antibody. Immunoblotting of 10 μg/lane ECM fractions of UUO kidney extracts from mice subcutaneously administered with saline, PEG, FUD or PEG-FUD. The first two lanes contain purified FUD (1000 ng) and PEG-FUD (0.005 ng). Blot was reacted with 0.7 μg/ml rabbit polyclonal antibody generated to KLH-C-terminal half of FUD. Loading control for ECM fractions was achieved with rabbit-anti Histone3 (1:10000). Molecular weight markers are depicted to the left of the blot. Primary antibodies were followed by a 1:10000 dilution of anti-rabbit-HRP-IgG and chemiluminescence. Mouse ID numbers are depicted above corresponding lane. Note the polyclonal anti-FUD 1) recognizes PEGylated FUD much better than unconjugated FUD both in purified form and tissue extracts; and 2) is highly specific for the two bands associated with PEG-FUD without spurious reactivity towards other proteins in kidney tissues; 3) no PEG-FUD fragmentation was detected.

Because peptide therapeutics are often degraded in the circulation or filtered through the kidney before they have a chance to act, we wished to determine whether PEG-FUD reached the kidneys and if so, whether it remained intact. As shown in Fig 3, PEG-FUD was identified in the ECM fractions of kidney extracts, recognized by an anti-FUD affinity-purified IgG. Note similar levels of intact PEG-FUD in kidneys of five different mice. Antibody recognition of PEG-FUD was specific with no spurious or background bands in the ECM fraction of kidney tissues from mice that were not administered PEG-FUD.

Interestingly, the antibody did not react with FUD in tissues as avidly as with PEG-FUD, as shown in Fig 3. Incubation with anti-histone3 antibodies demonstrates similar amounts of protein extracts of UUO kidneys from mice treated with FUD. Both Fig 3 and S1 Fig, demonstrate differential antibody reactivity towards purified FUD and PEG-FUD. This is not entirely surprising because the antibody was generated against KLH-conjugated FUD and affinity purified on a peptide-agarose column, thus reactivity was elicited and enriched for affinity towards a conjugated version of FUD. The affinity purified antibody recognized 500 pg PEG-FUD but only faintly recognized 500 ng of FUD (S1 Fig). Similarly, faint reactivity to 1 μg purified FUD is shown in Fig 3. As shown in S2 Fig, analysis by SDS-PAGE of purified peptides, loaded at 5 μg/lane to allow detection with Coomasie Blue, demonstrated comparable protein levels for FUD and PEG-FUD. PEGylated peptides appear as diffuse bands due to polydispersity properties of the PEG moiety [43]. This confirms that the anti-FUD antibodies recognize PEG-FUD better than FUD as an intrinsic property of the antibodies and not because of differences in protein concentration assessment or loading onto SDS-PAGE gels.

S3 Fig shows the sensitivity of the antibody for purified PEG-FUD under our Western blotting conditions was as low as 5 pg, and the level of PEG-FUD present in 10 μg ECM fractions of UUO kidneys was comparable to 500 ng PEG-FUD. Extrapolation suggests levels approximating 50 ng PEG-FUD per mg of kidney protein. Note consistency of similar levels of PEG-FUD in kidney extracts of five different mice. S4 Fig shows Western blotting for PEG-FUD in different kidney fractions. Distribution of PEG-FUD within the kidney suggested a tendency towards enrichment in UUO vs contralateral kidneys, but with similar levels in the ECM (pellets) compared to lysate fractions (S4 Fig). Normalization with loading controls for this blot was achieved with proteins stained with Ponceau Red [44] to allow comparison of pellets vs lysates. PEG-FUD was present in both UUO and contralateral kidneys in intact form. We also detected intact PEG-FUD at consistent levels in plasma (diluted 1:1000) of 5 different mice. Extrapolating from varying amounts of purified PEG-FUD, the level of PEG-FUD in plasma may approximate 50 μg/ml (S5 Fig).

### Assessment of Fibronectin deposition in kidney ECM

**Fig 4:**
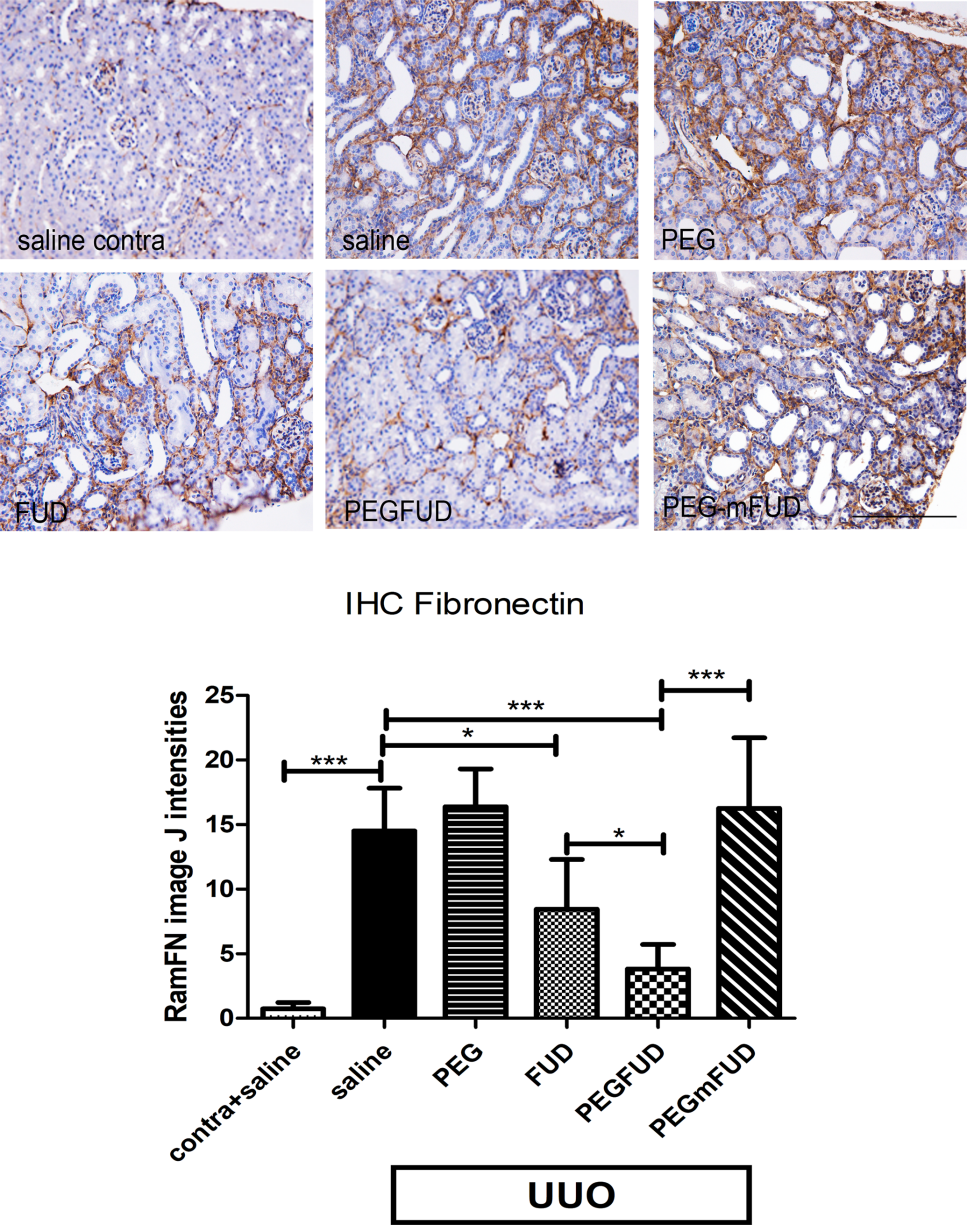
IHC shows the increased level of fibronectin found in the interstitium of UUO compared to contralateral kidneys was significantly reduced with PEG-FUD treatment. Representative images from the central cortex of 4 μm kidney sections stained with rabbit polyclonal antibody to fibronectin IgG (RamFN) at 0.5 μg/ml. UUO and contralateral kidneys were stained simultaneously. Except for the contralateral kidney of saline treated mice shown in top left panel, all other images are from UUO kidneys for comparison of treatment with saline, PEG, FUD, PEG-FUD and PEG-mFUD. Bar = 200 μm. Quantitation of staining was performed using Image J and the mean of six images per treatment per cohort +/- SD is graphed. Significance is denoted as *** p<0.001; **p<0.01, *p<0.05.

To determine whether FUD and PEG-FUD inhibited fibronectin deposition in kidneys of UUO-treated mice, we assessed fibronectin at the protein level, using immunohistochemistry and immunoblots of kidney extracts utilizing a previously described polyclonal antibody to mouse fibronectin [36]. Shown in Fig 4 are representative images from the cortical regions of kidneys from mice treated with saline, PEG, FUD, PEG-FUD or PEG-mFUD. As expected, fibronectin staining was detected in interstitial spaces and was greatly increased in the UUO kidney. The graph in Fig 4 shows quantitation of this staining using Image J software [45] and Student t-Test statistical comparison analysis. Fibronectin was significantly increased (~14-fold) in UUO compared to contralateral kidneys. FUD and PEG-FUD significantly decreased fibronectin deposition by 40% and 70%, respectively. Neither PEG nor PEG-mFUD decreased fibronectin.

**Fig 5:**
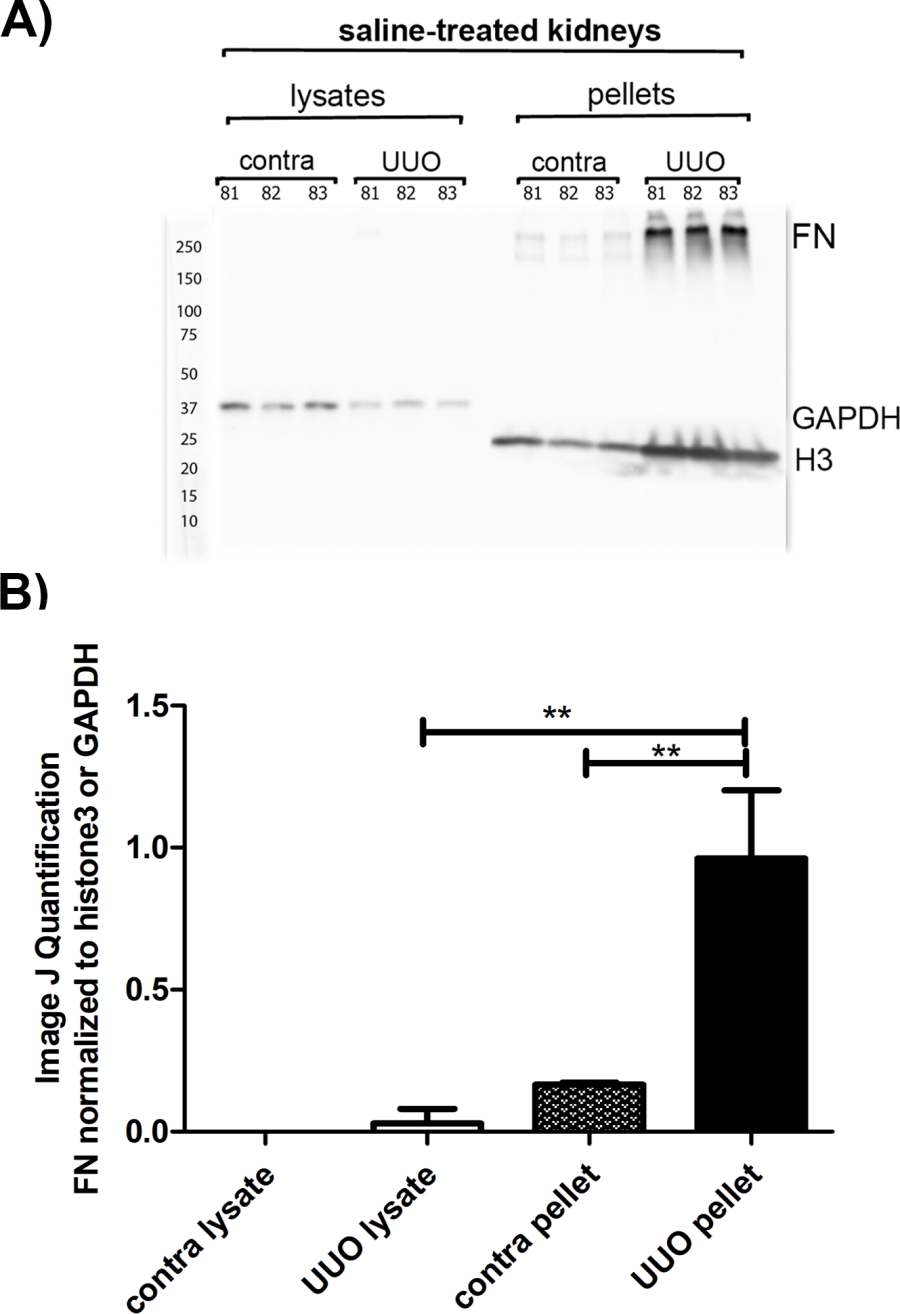
Fibronectin is enriched in the ECM fraction of UUO kidneys from mice treated with saline. Distribution of fibronectin into different tissue compartments was analyzed by fractionation of UUO and contralateral kidneys from three different mice treated with saline (mouse IDs above lanes) into DOC-insoluble (pellets) corresponding largely to ECM and DOC-soluble (lysates) corresponding to cytosolic and membrane proteins (10 μg/lane). A) Western blotting was carried out incubating the blot with RamFN IgG at 2 ng/ml followed by anti-rabbit-HRP IgG at 1:10000. Loading controls were GAPDH for lysate fractions and histone 3 (H3) for DOC-insoluble fractions. Molecular weight markers are depicted to the left of the blot. B) Quantitation of bands was carried out using Image J. Mouse ID numbers are depicted above corresponding lane. The means of fibronectin (FN) bands normalized to loading control bands from three mice +/- SD are presented. Significance is denoted as ** p< 0.01, as per Student t-Test analysis.

To corroborate this finding and determine whether the deposited fibronectin was intact or fragmented due to treatment, we extracted kidney tissues separating them into ECM and cytosolic/membrane fractions. It has been previously demonstrated in vitro that fibrillar fibronectin is typically enriched in the detergent-insoluble fraction (ECM fraction) of DOC extracted fibroblast monolayers [38], but to our knowledge, this has not been shown for kidney tissues. The cortical regions of kidneys were extracted with lysis buffer containing 1% DOC as described above. Following centrifugation, the soluble fraction was removed, termed “lysates” in this study, and the insoluble ECM fraction, termed “pellets” was solubilized with SDS-PAGE-loading buffer containing 4 M urea. Fig 5 shows the distribution of fibronectin in extracts from three contralateral kidneys compared to the corresponding UUO extracts. As shown in Fig 5A, RamFN recognized the 250 kDa band expected for fibronectin in the pellets (ECM) from both UUO and contralateral kidney extracts under reducing conditions. Next to nil fibronectin was detected in the lysates (cytosolic/membrane) from either the UUO or contralateral kidney extracts. Surprisingly, no degradation of fibronectin was apparent in UUO tissues, despite the remodeling and proteolysis expected in tissue undergoing inflammation and oxidative stress [31, 46, 47]. Histone3 and GAPDH were used as loading control molecules for normalizing fibronectin band intensities in pellets and lysates, respectively. Normalized values of fibronectin clearly indicated a significant enrichment of fibronectin in the ECM fraction of UUO compared to contralateral kidneys, as shown in Fig 5B.

**Fig 6:**
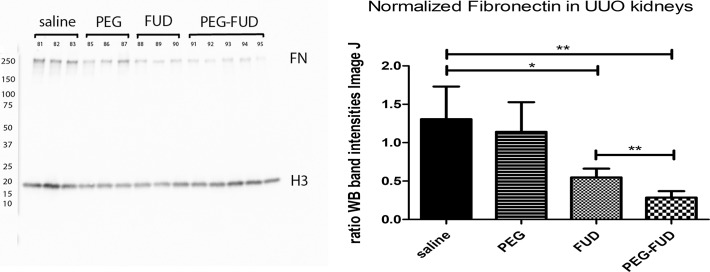
Immunoblotting shows PEG-FUD significantly decreased fibronectin without degradation in ECM fractions of UUO kidneys. DOC-insoluble fractions from UUO kidneys (10 μg/lane) from mice treated with saline, PEG, FUD or PEG-FUD were immunoblotted using RamFN IgG at 2 ng/ml and anti-histone3 at 1:10000, followed by anti-rabbit-HRP IgG at 1:10000. Quantitation of fibronectin and histone 3 band intensities was carried out using Image J. The means of fibronectin (FN) bands normalized to H3 from 3 or 5 mice (as indicated) +/- SD are presented. Mouse ID numbers are depicted above corresponding lane. Significance is denoted as * p < 0.05; ** p < 0.01, as per Student t-Test analysis.

Having shown that most FN is present in the ECM fraction of UUO kidneys, we focused on those fractions for further analyses. Shown in Fig 6A is an immunoblot of ECM fractions from UUO kidneys of mice treated with saline, PEG, FUD or PEG-FUD reacted with anti-fibronectin antibody. Again, fibronectin was recognized as a 250 kDa band and histone3 at 17 kDa was utilized as a loading control. Relative quantitation of the intensities of fibronectin and histone3 bands using Image J is presented as normalized values shown in Fig 6B. Compared to saline-treated UUOs, FUD and PEG-FUD decreased fibronectin by ~60% and ~80%, respectively. PEG did not decrease fibronectin significantly. Again, no proteolytic fragments were detected with the polyclonal antibody to fibronectin, suggesting that inhibition of fibronectin deposition into kidney matrices does not render fibronectin susceptible to proteolysis. This was also the case for fibronectin in plasma (S6 Fig).

### Quantitative assessment of histological collagen and leukocytes

**Fig 7:**
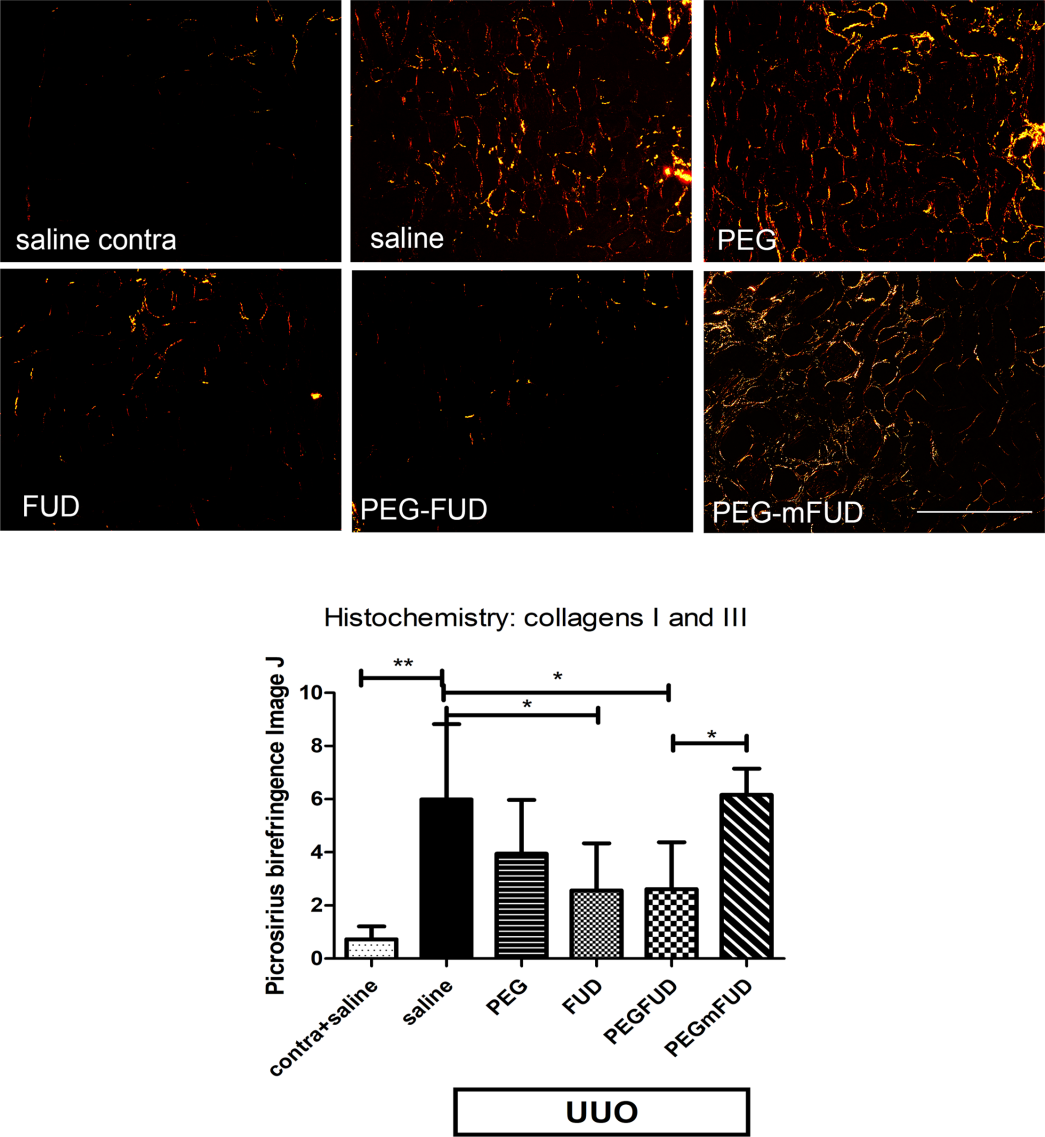
Collagens increased in the interstitium of UUO kidneys is significantly reduced by FUD and PEG-FUD. Representative images from the central cortex of 4 μm kidney sections stained with picrosirius red, which stains primarily collagens I and III. Birefringence elicited from exposure to polarized light was imaged. UUO and contralateral kidneys were stained simultaneously. Except for the contralateral kidney of saline treated mice, all other images are from UUO kidneys for comparison of treatment with saline, PEG, FUD, PEG-FUD and PEG-mFUD. Bar = 200 μm. Quantitation of staining was performed using Image J and the mean of six images per treatment per cohort +/- SD was graphed. Significance is denoted as * p < 0.05; ** p<0.01, as per Student t-Test analysis.

Once it was clear that PEG-FUD decreased fibronectin in the kidney, we sought to determine possible effects on collagen deposition and leukocyte infiltration. Fibronectin has been shown to modulate and, in some microenvironments, control the deposition of collagens, structural proteins responsible for scar formation and increased tissue density associated with fibrosis. We detected collagens I and III by illuminating picrosirius red-stained tissue sections with polarized light. The resulting birefringence associated with the collagen fibers was imaged and quantified using Image J [34]. Fig 7 shows representative images from kidney sections sequential to those described above for fibronectin. Contralateral kidneys of mice treated with saline show negligible collagen in the cortical interstitium (top, left panel). The corresponding UUO section (top central panel) shows increased collagen as expected for UUO-treated kidneys [11, 14, 48]. Relative quantitation performed with Image J shows greater variability in collagen detection for most of the treatment groups than that observed for fibronectin. Nevertheless, there was a significant decrease in collagens in kidney sections of mice treated with FUD or PEG-FUD of about 50% and no significant effect with PEG or PEG-mFUD.

**Fig 8:**
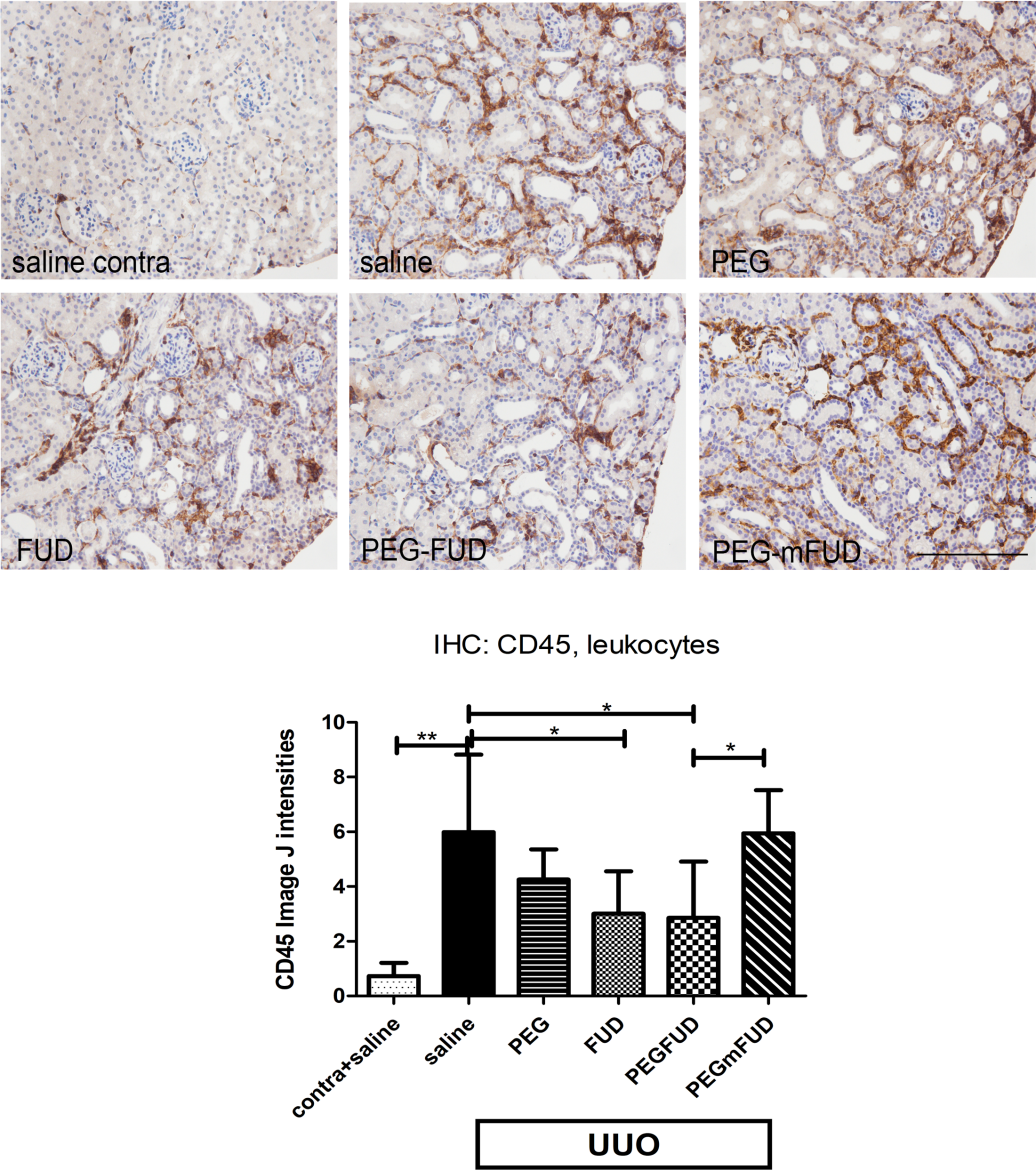
IHC shows the increased level of CD45+ leukocytes found primarily in the interstitium of UUO kidneys was significantly reduced with FUD and PEG-FUD treatment. Representative images from the central cortex of 4 μm kidney sections stained with rat anti-mouse CD45 at 2.5 μg/ml. UUO and contralateral kidneys were stained simultaneously. Except for the contralateral kidney of saline treated mice, all other images are from UUO kidneys for comparison of treatment with saline, PEG, FUD, PEG-FUD and PEG-mFUD. Bar = 200 μm. Quantitation of staining was performed using Image J and the mean of six images per treatment per cohort +/- SD is graphed. Significance is denoted as * p< 0.05; ** p<0.001.

It was reported in other studies that a decrease in fibronectin was associated with decreased leukocyte infiltration [11, 17, 49]. The representative images shown in Fig 8 indicated a low level of leukocytes in the contralateral kidneys treated with saline, which was also representative of contralateral kidneys in all treatment groups. The corresponding UUO kidney section shows clear infiltration of leukocytes into the interstitium. Quantitation of staining by Image J, shows a 6-fold increase in CD45 staining in UUO compared to contralateral kidneys. There was a significant decrease in CD45 staining of ~50% with both FUD and PEG-FUD, and variability similar to that observed for collagen. There was no significant change with PEG or PEG-mFUD.

### Qualitative histological assessment

**Fig 9:**
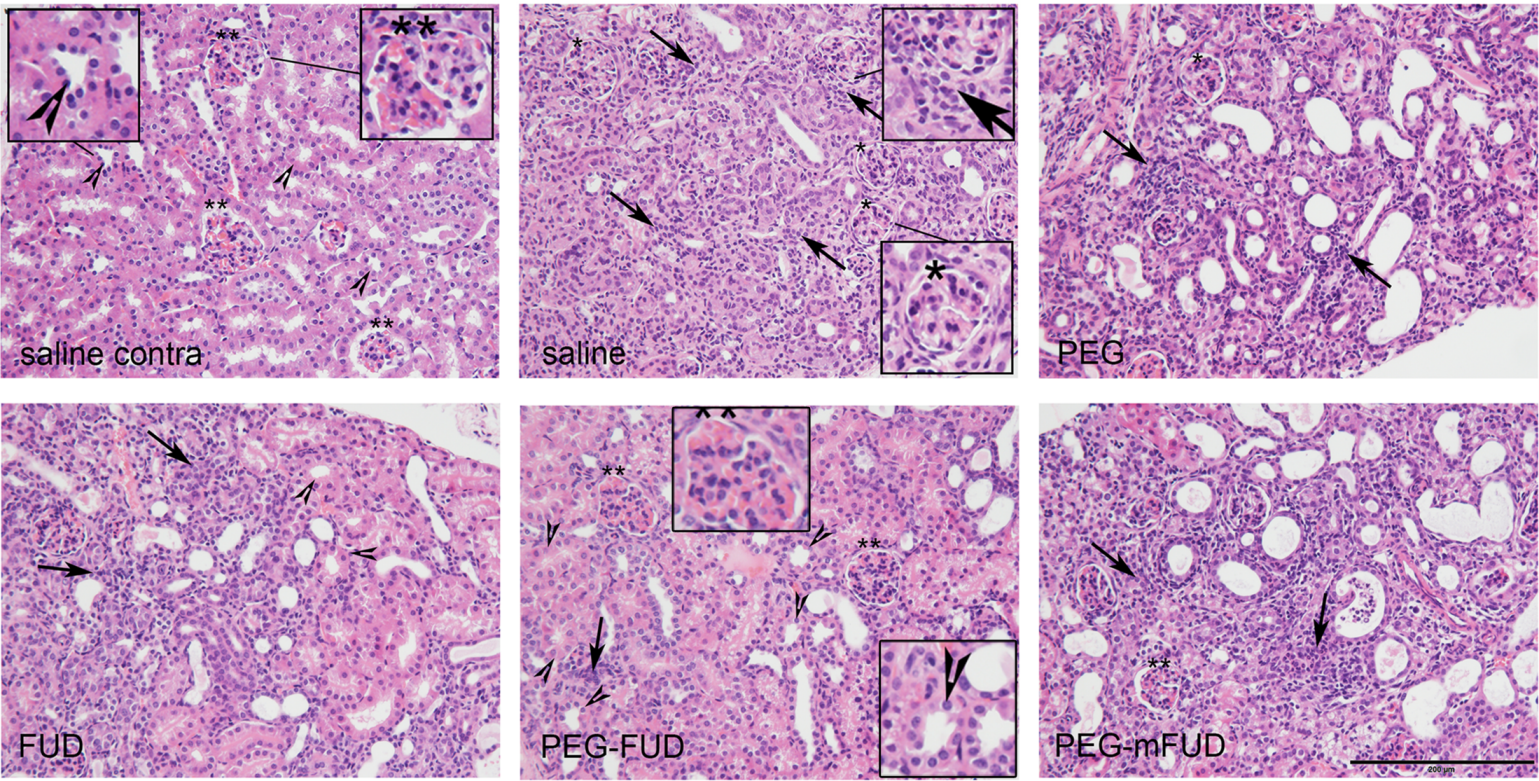
Hematoxylin-eosin staining of UUO kidneys shows improved histology with PEG-FUD treatment. Representative images from the central cortex of 4 μm kidney sections stained with H&E. UUO and contralateral kidneys were stained simultaneously. Except for the contralateral kidney of saline treated mice, all other images are from UUO kidneys for comparison of treatment with saline, PEG, FUD, PEG-FUD and PEG-mFUD. Eosin staining was more prominent in the saline treated contralateral kidney, was associated with intact proximal tubules (arrowheads) and with defined glomeruli containing red blood cells (double asterisks). Hematoxilin staining associated with infiltrating cells, interstitial cell proliferation (arrows) and less defined glomeruli lacking red blood cells (single asterisk) were more prominent in the saline, PEG or PEG-mFUD-treated UUO kidneys. Diminished hematoxylin, increased eosin staining associated with tubular structures, and vascularized glomeruli similar to the contralateral kidney, were more apparent in the FUD and, to a greater extent, the PEG-FUD-treated kidneys, suggesting improvement in tubular and glomerular health. Bar = 100 μm.

To investigate possible effects on viability of proximal tubules, sections sequential to those described above were stained for H&E. Fig 9 shows representative images of H&E-stained sections from the central cortical regions of a contralateral kidney treated with saline (saline contra, left upper panel) in comparison with sections from UUO-treated kidneys (all other panels). Comparison of the contralateral and UUO kidneys from saline-treated mice indicates strong eosinophilic staining in the healthy kidney, which is lost with UUO treatment. Increased hematoxylin staining in the UUO kidneys is largely due to infiltration of leukocytes, loss of proximal tubules and proliferation of fibroblasts [32]. Comparing treatments of UUO kidneys, we observed greater eosinophilic staining with FUD and PEG-FUD compared to saline; and similar to the contralateral kidney, clearer tubular preservation and vascularized glomeruli with PEG-FUD. Treatment with either PEG or PEG-mFUD did not increase eosinophilic staining and showed notably damaged tubules.

## Discussion

We set out to ascertain whether the PEGylated form of FUD could reach injured kidneys and inhibit fibronectin deposition into the interstitium of UUO kidneys. In addition, we sought to determine if inhibition of fibronectin assembly would result in inhibition of kidney fibrosis, as ascertained by interstitial collagen deposition and leukocyte infiltration [3, 6, 11]. Following subcutaneous administration, PEG-FUD was found intact in kidney extracts as per Western blotting analysis with some enrichment in UUO compared to contralateral kidneys. UUO kidneys presented the expected increase in fibronectin, collagen and CD45^+^-leukocytes. Administration of PEG-FUD for 7 days, resulted in a significant decrease in fibronectin, collagen and leukocytes in UUO-treated kidneys. Qualitative evaluation of H&E-stained kidney sections suggested improvement in tubular atrophy with PEG-FUD treatment.

Conjugation with PEG is a well-established strategy for mitigation of challenges involved in the delivery of therapeutic peptides. At least ten PEGylated biologics have been FDA approved and are on the U.S. market today [50]. Some benefits of PEGylation include improved circulation time due to a reduction in immunogenicity and proteolytic degradation [21]. The principal cause of this improvement is the contribution of the PEG moiety’s large hydrodynamic radius to the polymer-drug conjugate [50]. PEGylation often impedes or diminishes the interaction of the PEGylated molecule with its ligand [51]. However, PEG-FUD retained activity and inhibited the incorporation of fibronectin into the ECM of fibroblasts and proximal tubular epithelial cells with IC50s similar to unconjugated FUD without affecting cell viability, as demonstrated previously for FUD [8, 16, 42]. Thus, PEGylation did not affect FUD affinity for fibronectin and is expected to decrease both blood-derived and locally derived EDA+- fibronectin incorporation into tissues without deleterious effects on cell viability.

In vivo, PEG-FUD significantly decreased fibronectin in UUO kidneys, apparently to a greater extent than FUD itself. Because the intrinsic properties of our anti-FUD antibodies do not allow for detection of unconjugated FUD, we could only detect PEG-FUD and not FUD in kidney extracts. We attribute the preference of the anti-FUD antibodies for PEG-FUD to the fact that the antiserum was raised against FUD conjugated to KLH, which likely rendered the peptide into a conformation similar to that of PEGylated FUD. In any case, we cannot ascertain whether PEG-FUD is a more potent inhibitor of fibronectin deposition in vivo, or is retained better in diseased kidneys than FUD. Regardless, it is clear that PEG-FUD is a specific, potent inhibitor of fibronectin deposition into the interstitium of UUO kidneys.

The significant decrease in fibronectin precludes any concern that PEG-FUD would form complexes with fibronectin that would deposit in the interstitium and harm kidney tubules. If such complexes were present, there would have been increased staining for fibronectin, likely even higher than expected for UUO kidneys [4, 31, 52]. Instead, we observed the opposite, decreased fibronectin with PEG-FUD treatment. In addition, using PEG-FUD to inhibit the deposition of fibronectin into the ECM did not cause any detectable levels of soluble fibronectin degradation, since intact fibronectin was detected in the kidney fractions, as well as in plasma. Indeed, the binding of PEG-FUD to fibronectin which, under in vitro conditions, is considerably tight (Kd~10 nM) [22], may actually protect at least the N-terminal 70 kDa fragment from degradation. That fibronectin is intact is of importance because fibronectin fragments are known to promote remodeling and pathological degeneration in some tissues [53].

Inhibiting fibronectin fibrillogenesis with PEG-FUD showed that fibronectin plays two important roles in the progression of kidney fibrosis. First, fibronectin serves a pivotal role in the deposition of collagens, as demonstrated in this study and the studies of others in vitro [7, 8] and in vivo [17–19]. When we examined the effects of decreased fibronectin on the deposition of collagens I and III, as detected by picrosirius staining [34], we detected a significant decrease in collagens with treatment of FUD or PEG-FUD but not PEG or PEG-mFUD. Second, fibronectin has been associated with infiltrating leukocytes in a variety of conditions, serving as adhesive substratum and/or as ligand for Toll-like receptors [11, 49, 54–56]. Similarly to collagen inhibition, FUD or PEG-FUD treatment was associated with a decrease in leukocyte infiltration in UUO kidneys suggesting that fibronectin plays a role in the inflammatory response in the kidney. Interestingly, the contralateral kidneys showed negligible levels of leukocyte infiltration with all treatments, suggesting no detectable immunoreactivity towards the peptides. Finally, H&E staining of UUO kidneys from mice treated with PEG-FUD, shows diminished tubular atrophy, possible improved glomerular vascularization,and confirms the reduction in leukocyte infiltration. However, because the contralateral kidney remains intact in the UUO model, renal function is not affected [57]. Therefore, whether PEG-FUD improves renal function will necessitate future testing in a kidney disease model that affects both kidneys, such as the ischemia reperfusion injury, subtotal 5/6 nephrectomy [57, 58], or chronic allograft nephropathy [59].

An important consideration is that fibronectin is intrinsic to wound healing mechanisms, forming part of the temporary matrix necessary for wound closure [29]. Thus, a concern is that inhibiting fibronectin assembly may delay tubular regenerative capacity. These concerns may not be merited as mice with a conditional knock-out for plasma fibronectin, which constitutes about 50% of fibronectin deposited in tissues [40], show negligible hemostasis pathology [60]. In addition, people with about half the normal levels of circulating fibronectin show no hemostasis abnormalities [61]. Nevertheless, we were attentive to this issue and adjusted the timing of PEG-FUD delivery to target excess deposition of ECM while still allowing the healing process to occur and thus started administration three days post-UUO surgery. As noted, we detected the opposite in our pilot study, that is, protection from tubular atrophy with PEG-FUD and FUD. It will be incumbent to future work in this area to assess various timing regimes and dosage to optimize efficacy of fibronectin deposition inhibitors. It will also be interesting to test PEG conjugates testing the possible benefits of different size of PEG chains, such as 10 kDa or 40 kDa to ascertain best kidney retention and fibronectin inhibitory activity.

There are a number therapeutics currently being tested in clinical trials that inhibit fibrosis such as Pirfenidone, Avosentan, THR-184, monoclonal antibodies to TGF-β or CTGF [62]. In addition, drugs currently used as therapy for kidney disease, such as inhibitors of the renin-angiotensin system, directly or indirectly inhibit fibrosis [63, 64]. However, these therapeutics target molecules with pleiotropic activities that likely perturb pathways fundamental to normal kidney physiology, and thereby can cause undesirable side effects. Future studies, testing PEG-FUD, optimized for PEG size and dose in models of kidney disease amenable to renal function assessment, will help ascertain whether inhibition of fibrosis associated with specific decrease of fibronectin deposition translates into improved renal function. Excessive ECM is common to kidney diseases, regardless of etiology [4, 65, 66]. Indeed, fibrotic diseases in general are responsible for 40% of morbidity in the developed world [67]. PEG-FUD may then become a novel potential anti-fibrotic therapeutic, specifically targeting excessive ECM deposition in a variety of diseases.

## Supporting information

S1 Fig Rabbit polyclonal anti-FUD IgG recognizes PEG-FUD but not FUD by immunoblotting. Purified PEG-FUD at 0.005, 0.05, 0.5, 5 and 50 ng per lane, and FUD at 5, 50 and 500 ng per lane were separated on a 4–20% gradient gel and immunoblotted with rabbit anti-FUD IgG at 0.7 μg/ml, followed by HRP-conjugated anti-rabbit IgG at 1:10000. Molecular weight markers are depicted to the left of the blot. PEG-FUD migrates primarily ~ 50 kDa mark with a less prominent band at 100 kDa. Recognition of PEG-FUD in the left blot was of high avidity with a sensitivity of 5 pg; band intensity correlated with amount of protein loaded per lane. The blot to the right shows recognition of unconjugated FUD was almost nil. PEG-FUD at 0.5 ng was also run in this blot as a positive control.
(TIF)

S2 Fig SDS-PAGE analysis of purified peptides. Purified FUD, mFUD, PEG-FUD and PEG-mFUD were loaded onto a 4–20% polyacrylamide gel at 5 μg/lane, run as per standard conditions and stained with Coomasie Brilliant Blue. Molecular weight standards are depicted to the left of the gel. The molecular weights of FUD and of PEG-FUD are ~7 and ~ 27 kDa, respectively as determined by mass spectrometry [22]. However, on SDS-PAGE, both migrate close to the 50 kDa marker. It is well recognized that short peptides (<10 kDa), can migrate anomalously on SDS-PAGE [68], depending on their axial ratios or hydrophobic amino acid content [69, 70]. In addition, PEG moieties are polydisperse and may also alter the electrophoretic mobility of its peptide conjugates [71]. In the PEGylated peptides, there is a fainter band at 100 kDa, which may represent dimerization of the conjugate. Dimerization may occur upon handling or freezing and thawing of the conjugated peptide, but upon purification there was no dimerization detected by HPLC or mass spectrometry.
(TIF)

S3 Fig Levels of PEG-FUD in ECM fractions of UUO kidneys were consistent and approximate 50 ng/mg kidney tissue. Immunoblot of purified PEG-FUD at 0.005, 0.05, 0.5 and 5 ng compared to 10 μg pellet fractions of UUO kidneys from 5 mice administered PEG-FUD. Loading control was histone 3. Note consistency in levels of PEG-FUD in UUO ECM tissue fractions of 3 different mice. The intensity of the 50 kDa PEG-FUD band was deemed most similar to 0.5 ng of purified PEG-FUD. Thus, 0.5 ng/10 μg tissue protein was extrapolated to estimate 50 ng PEG-FUD per mg kidney tissue. Mouse ID numbers are depicted above corresponding lane. Molecular weight markers are depicted to the left of the blot.
(TIF)

S4 Fig PEG-FUD was detected in UUO and contralateral kidneys and in both ECM and cytosolic/membrane fractions. Immunoblot of ECM (pellets) and cytosolic/membrane (lysates) at 10 μg/lane from kidneys of mice treated with PEG-FUD. Purified PEG-FUD at 0.5 ng/lane was run for reference. Molecular weight markers are depicted to the left of the blot. Quantitation of the 50 kDa PEG-FUD band was carried out using Image J and normalized to protein bands visible in the central region of the blot with Ponceau stain. The means of the normalized intensities are presented +/- SD showing a slight enrichment of PEG-FUD in UUO kidneys compared to contralateral. Mouse ID numbers are depicted above corresponding lane Significance is denoted as * p<0.05.
(TIF)

S5 Fig PEG-FUD was detected in intact form and circulated at consistent levels in plasma. Plasma was collected at harvest from mice receiving PEG-FUD and diluted to 1:1000; 10 μl were loaded per lane. Purified PEG-FUD at 0.05, 0.5 and 5 ng/lane were added for reference. The blot was reacted with rabbit-anti-FUD IgG at 0.7 μg/ml followed by HRP-conjugated anti-rabbit IgG at 1:10000. As in tissues, the levels of PEG-FUD in plasmas from 5 different mice were also consistent. Circulating PEG-FUD appeared intact and was similar in intensity to the 0.5 ng PEG-FUD reference which suggests a circulating level of ~ 50 μg/ml (50 ng per 10 μl loaded x 1000 dilution factor). Mouse ID numbers are depicted above corresponding lane. Molecular weight markers are depicted to the left of the blot.
(TIF)

S6 Fig Fibronectin was detected in intact form and was slightly elevated in the plasma of PEG-FUD treated mice. Plasma collected at harvest was diluted 1:1000 and 10 μl loaded per lane. Blot was reacted with rabbit polyclonal to fibronectin (RamFN) at 2 ng/ml, followed by HRP-conjugated anti-rabbit IgG at 1:10000. Mouse ID numbers are depicted above corresponding lane. Molecular weight markers are depicted to the left of the blot.
(TIF)
