# Supplementary figures and images for: PEGylated pUR4/FUD peptide inhibitor of fibronectin fibrillogenesis decreases fibrosis in murine Unilateral Ureteral Obstruction model of kidney disease
Source: PLoS One. 2018 Oct 24;13(10):e0205360. doi: 10.1371/journal.pone.0205360 (PMC6200241; doi:10.1371/journal.pone.0205360)

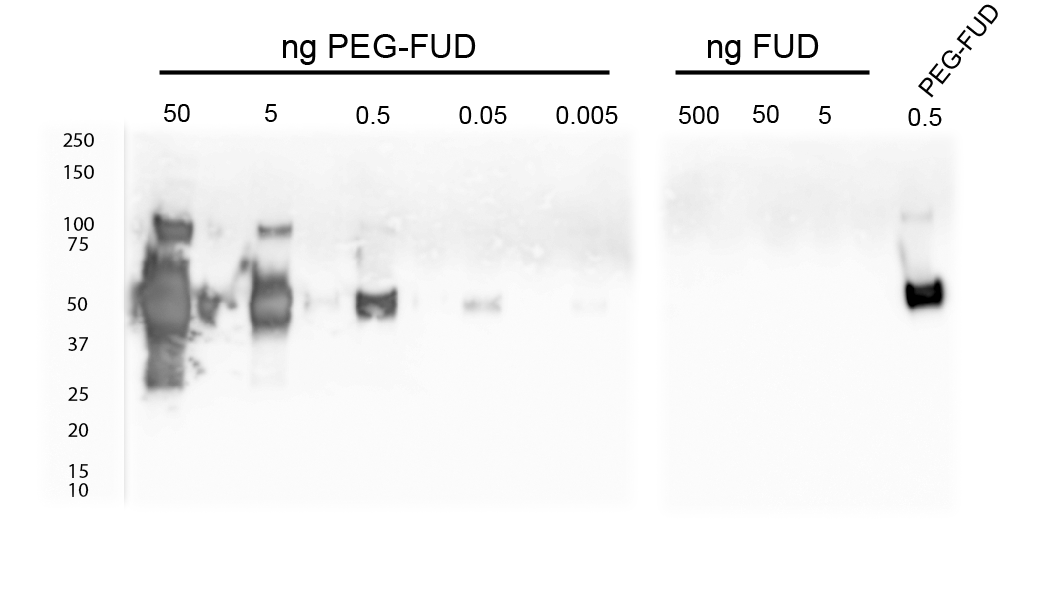

Supplement: S1 Fig — Purified PEG-FUD at 0.005, 0.05, 0.5, 5 and 50 ng per lane, and FUD at 5, 50 and 500 ng per lane were separated on a 4–20% gradient gel and immunoblotted with rabbit anti-FUD IgG at 0.7 μg/ml, followed by HRP-conjugated anti-rabbit IgG at 1:10000. Molecular weight markers are depicted to the left of the blot. PEG-FUD migrates primarily ~ 50 kDa mark with a less prominent band at 100 kDa. Recognition of PEG-FUD in the left blot was of high avidity with a sensitivity of 5 pg; band intensity correlated with amount of protein loaded per lane. The blot to the right shows recognition of unconjugated FUD was almost nil. PEG-FUD at 0.5 ng was also run in this blot as a positive control. (TIF) [file pone.0205360.s001.tif]

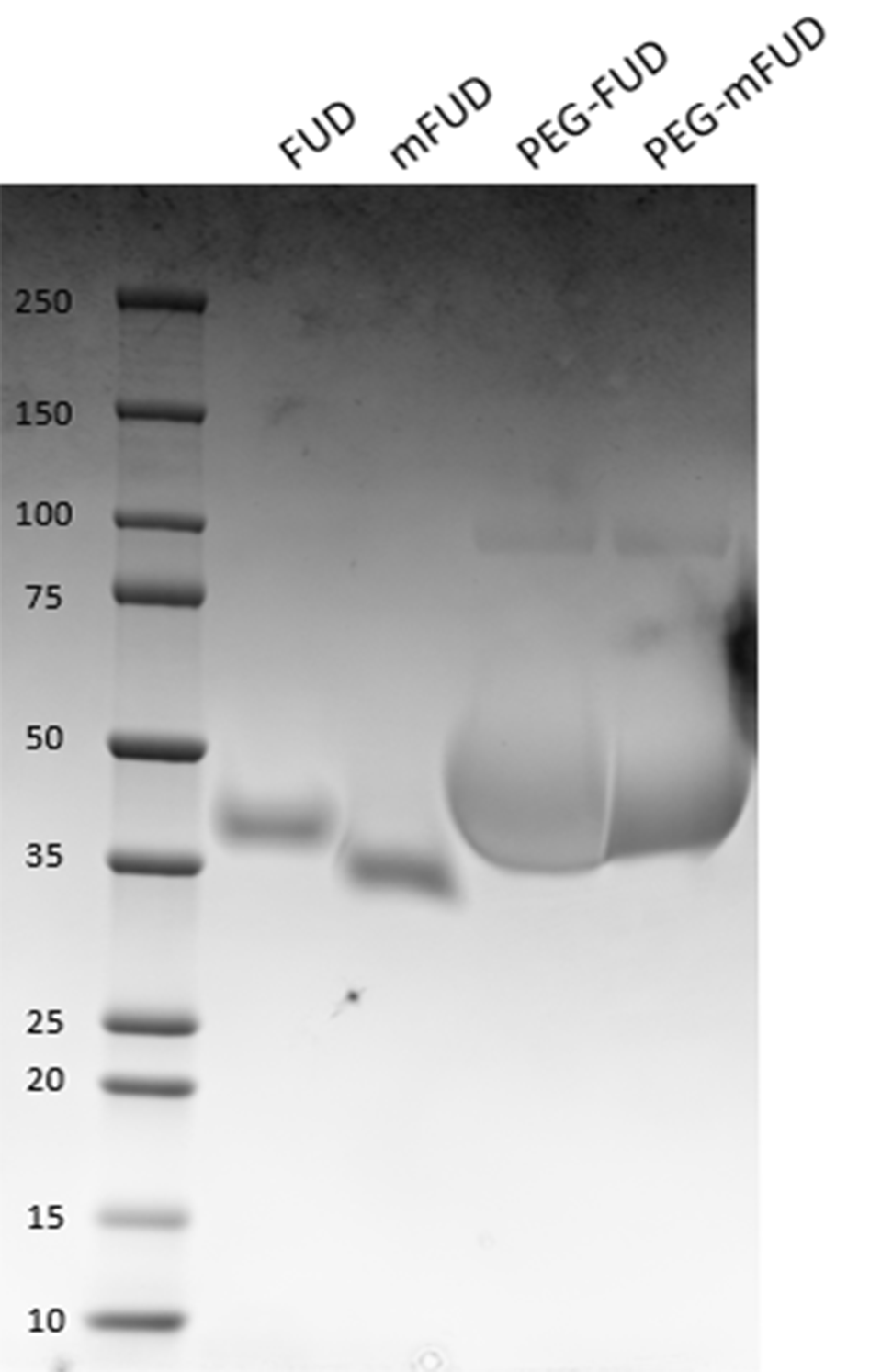

Supplement: S2 Fig — Purified FUD, mFUD, PEG-FUD and PEG-mFUD were loaded onto a 4–20% polyacrylamide gel at 5 μg/lane, run as per standard conditions and stained with Coomasie Brilliant Blue. Molecular weight standards are depicted to the left of the gel. The molecular weights of FUD and of PEG-FUD are ~7 and ~ 27 kDa, respectively as determined by mass spectrometry [22]. However, on SDS-PAGE, both migrate close to the 50 kDa marker. It is well recognized that short peptides (<10 kDa), can migrate anomalously on SDS-PAGE [68], depending on their axial ratios or hydrophobic amino acid content [69, 70]. In addition, PEG moieties are polydisperse and may also alter the electrophoretic mobility of its peptide conjugates [71]. In the PEGylated peptides, there is a fainter band at 100 kDa, which may represent dimerization of the conjugate. Dimerization may occur upon handling or freezing and thawing of the conjugated peptide, but upon purification there was no dimerization detected by HPLC or mass spectrometry. (TIF) [file pone.0205360.s002.tif]

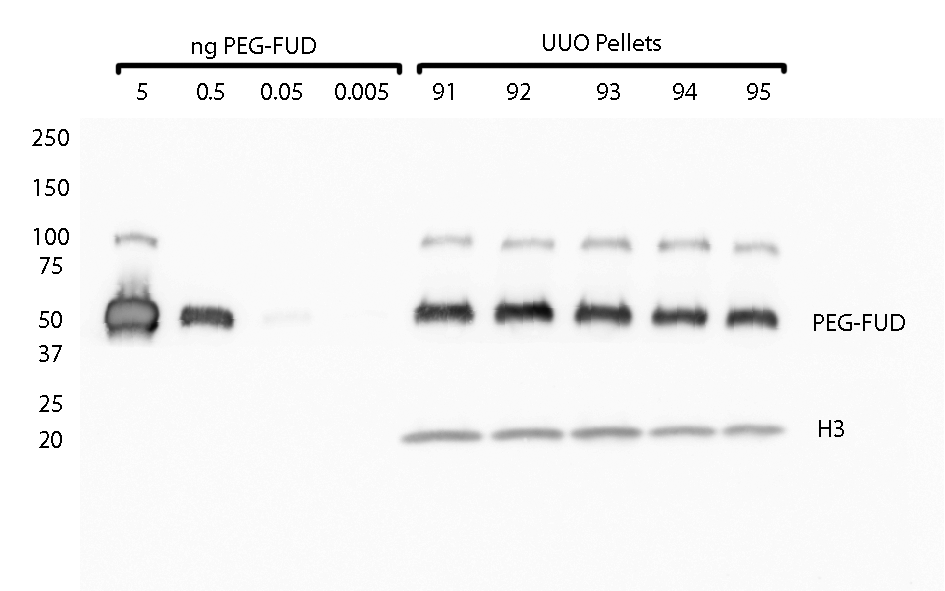

Supplement: S3 Fig — Immunoblot of purified PEG-FUD at 0.005, 0.05, 0.5 and 5 ng compared to 10 μg pellet fractions of UUO kidneys from 5 mice administered PEG-FUD. Loading control was histone 3. Note consistency in levels of PEG-FUD in UUO ECM tissue fractions of 3 different mice. The intensity of the 50 kDa PEG-FUD band was deemed most similar to 0.5 ng of purified PEG-FUD. Thus, 0.5 ng/10 μg tissue protein was extrapolated to estimate 50 ng PEG-FUD per mg kidney tissue. Mouse ID numbers are depicted above corresponding lane. Molecular weight markers are depicted to the left of the blot. (TIF) [file pone.0205360.s003.tif]

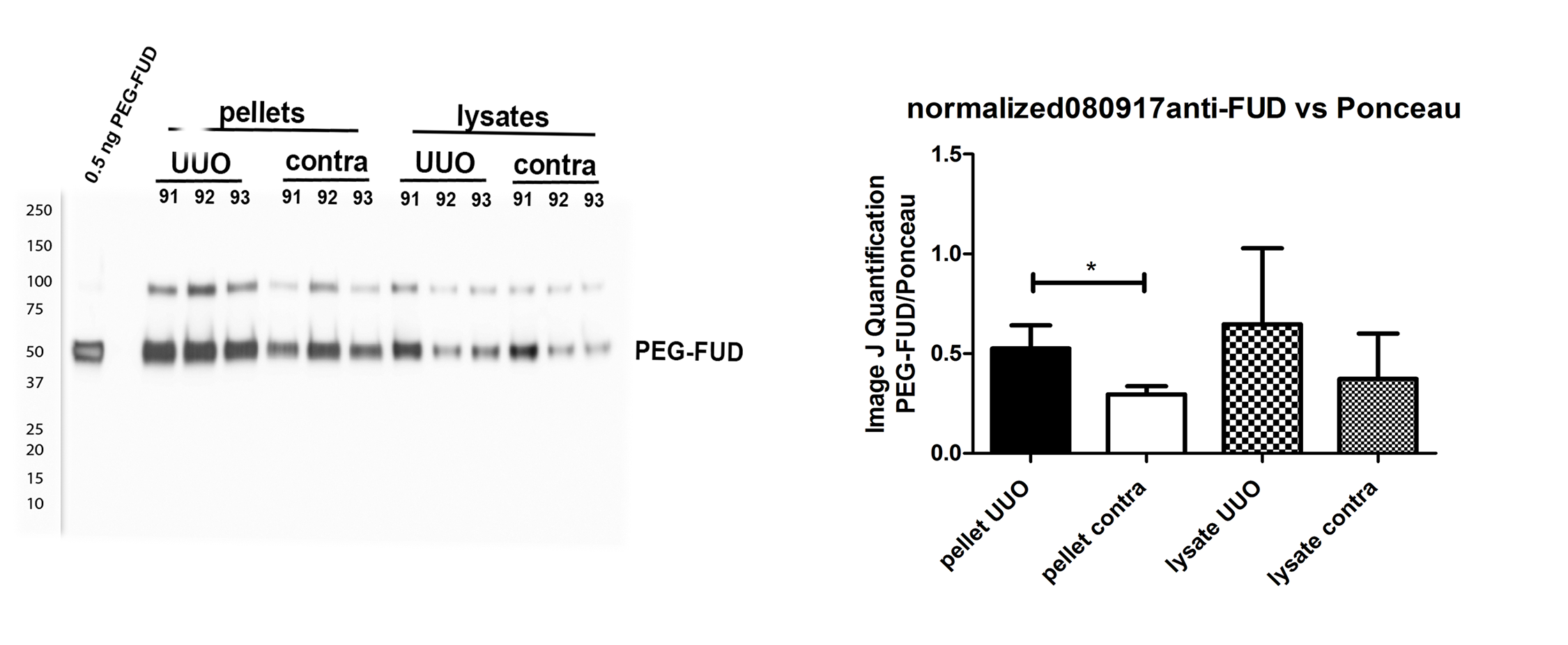

Supplement: S4 Fig — Immunoblot of ECM (pellets) and cytosolic/membrane (lysates) at 10 μg/lane from kidneys of mice treated with PEG-FUD. Purified PEG-FUD at 0.5 ng/lane was run for reference. Molecular weight markers are depicted to the left of the blot. Quantitation of the 50 kDa PEG-FUD band was carried out using Image J and normalized to protein bands visible in the central region of the blot with Ponceau stain. The means of the normalized intensities are presented +/- SD showing a slight enrichment of PEG-FUD in UUO kidneys compared to contralateral. Mouse ID numbers are depicted above corresponding lane Significance is denoted as * p<0.05. (TIF) [file pone.0205360.s004.tif]

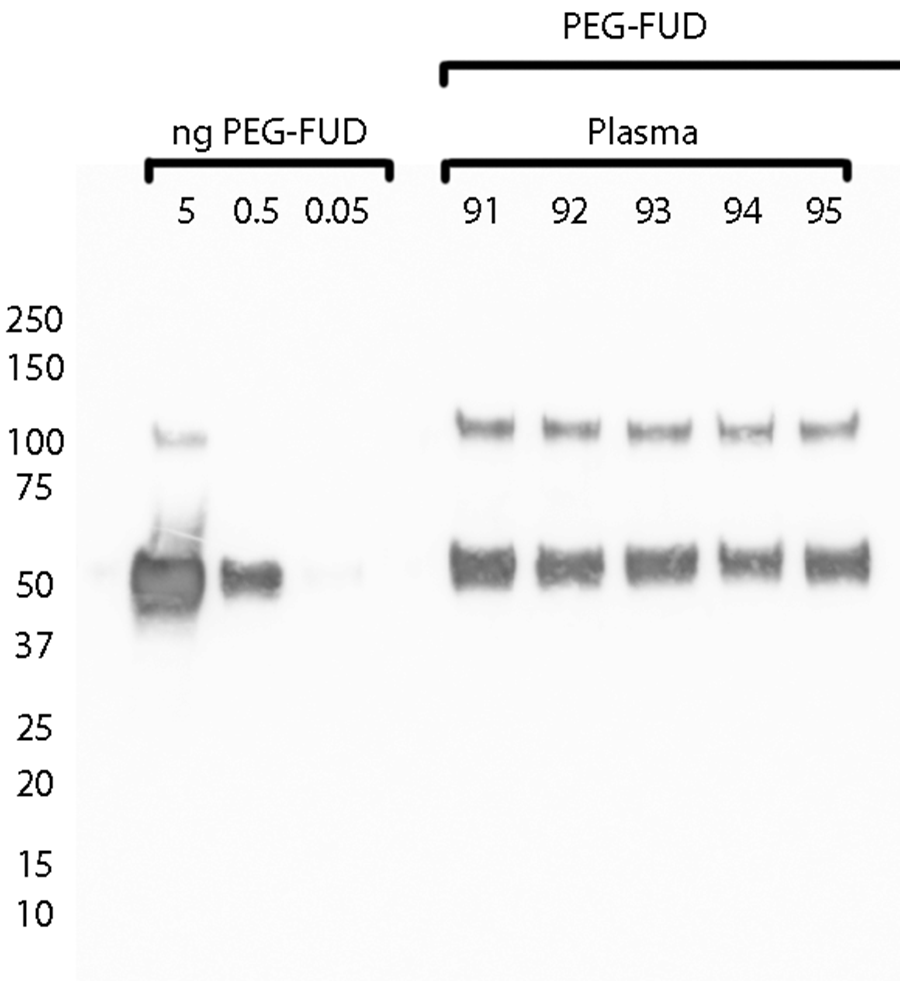

Supplement: S5 Fig — Plasma was collected at harvest from mice receiving PEG-FUD and diluted to 1:1000; 10 μl were loaded per lane. Purified PEG-FUD at 0.05, 0.5 and 5 ng/lane were added for reference. The blot was reacted with rabbit-anti-FUD IgG at 0.7 μg/ml followed by HRP-conjugated anti-rabbit IgG at 1:10000. As in tissues, the levels of PEG-FUD in plasmas from 5 different mice were also consistent. Circulating PEG-FUD appeared intact and was similar in intensity to the 0.5 ng PEG-FUD reference which suggests a circulating level of ~ 50 μg/ml (50 ng per 10 μl loaded x 1000 dilution factor). Mouse ID numbers are depicted above corresponding lane. Molecular weight markers are depicted to the left of the blot. (TIF) [file pone.0205360.s005.tif]

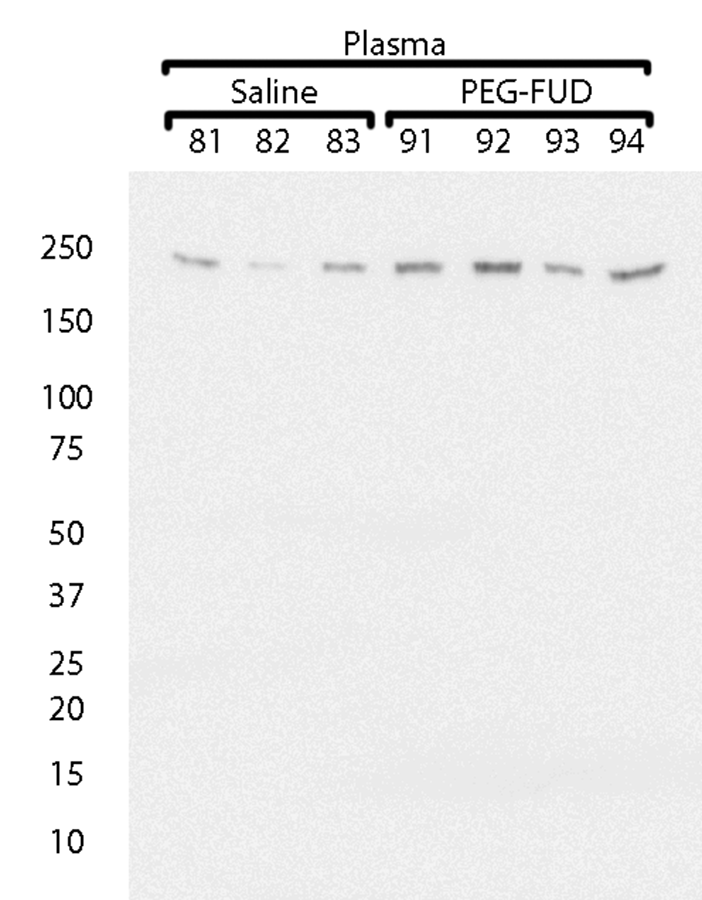

Supplement: S6 Fig — Plasma collected at harvest was diluted 1:1000 and 10 μl loaded per lane. Blot was reacted with rabbit polyclonal to fibronectin (RamFN) at 2 ng/ml, followed by HRP-conjugated anti-rabbit IgG at 1:10000. Mouse ID numbers are depicted above corresponding lane. Molecular weight markers are depicted to the left of the blot. (TIF) [file pone.0205360.s006.tif]
